# Seroprevalence and Risk Factors of *Toxocara* spp. in Dogs, Cats, and Their Owners in the City of Villavicencio, Colombia: Interaction With the “One Health” Approach

**DOI:** 10.1155/vmi/3517109

**Published:** 2026-05-31

**Authors:** María Alejandra Velásquez Peña, Chaira Liz Angelica Vasquez-Turriago, Adolfo Vásquez-Trujillo, Dumar Alexander Jaramillo-Hernández

**Affiliations:** ^1^ School of Animal Sciences, University of the Llanos, Villavicencio Meta, 1745, Colombia; ^2^ Doctoral Program in Agroscience, Faculty of Agricultural Sciences and Natural Resources, University of the Llanos, Villavicencio Meta, 1745, Colombia

**Keywords:** atopy, coprodiagnosis, geohelminth, immunoassay, IgG, toxocariasis

## Abstract

**Background:**

*Toxocara* spp. is one of the most neglected zoonotic soil‐transmitted helminths worldwide, causing toxocariasis in humans. The objective of this study was to determine the seroprevalence of *Toxocara* spp. in dogs, cats, and their owners, the coprodiagnostic incidence in dogs and cats, the risk factors, and association with atopy in owners of these pets.

**Methodology:**

A total of 70 dogs, 140 cats, and 118 owners were randomly selected. A blood sample was taken from each of them to determine anti‐*Toxocara* IgG by in‐house indirect ELISA using TES. Two fecal samples were taken from dogs and cats at 28‐day intervals, and the Kato–Katz technique was used for cumulative coproincidence. Prevalence ratio (PR) and relative risk (RR) tests were used to establish risk factors. The ISAAC III survey and skin prick test for aeroallergens were used to classify atopy in the pet owners.

**Results:**

The overall seroprevalence of anti‐*Toxocara* IgG in dogs and cats was 80% (95% confidence interval [CI]: 74.1%–84.8%), whereas in the owners it was 22.9% (95% CI: 16.2%–31.2%). The overall coproprevalence and coproincidence in dogs and cats were 47.6% (95% CI: 41%–54.3%) and 20% (95% CI: 13.6%–28.4%), respectively. Dogs and cats that go outside more than three times a day are more likely to contract *Toxocara* spp. (RR = 3.87). Owners who had a seropositive dog and cat at the same time were more likely to be seropositive for *Toxocara* spp (PR = 3.46). One of the risk factors for owners was having a dog or cat between 9 and 16 years of age (RR = 1.5) and sleeping in bed with these pets (RR = 1.93). There was no positive association between seropositivity for *Toxocara* spp. and atopy in owners (PR = 1.02).

**Conclusions:**

This is the first study in Colombia to determine *Toxocara* spp. seroprevalence in dogs and cats. The high cumulative coproincidence suggests significant exposure of dogs and cats to this parasite eggs in public spaces, which generates interest in public health and reinforces the need for a “One Health” approach.

## 1. Introduction

*Toxocara* spp. is a cosmopolitan parasite, a genus of helminth that mainly parasitizes canines and felines, with *Toxocara canis* and *Toxocara cati* being the most relevant species and with zoonotic potential for humans [1]. The life cycle of this parasite includes phases in the environment and paratenic hosts, facilitating its dispersion and perpetuation in the environment [2]. In domestic dogs and cats, infection by *Toxocara* spp. can generate diverse clinical manifestations that can range from mild gastrointestinal and respiratory symptoms (such as coughing due to larval migration in the lungs) to serious conditions such as malnutrition and intestinal obstructions [3]. Although most infections tend to be asymptomatic, animals can be reservoirs of the disease, implying a significant public health risk, especially in communities in which hygiene is poor and correlated with situations of poverty [4, 5].

Adult *T. canis* and *T. cati* develop their reproductive phase in the intestine of dogs and cats, which are their definitive hosts. It is noteworthy that a single adult female can lay up to 200,000 eggs per day, which are excreted in the feces [1, 6]. Transmission of *Toxocara* spp. can be vertical in pregnant dogs via transplacental and/or transmammary routes, whereas in pregnant cats, it is only transmammary. Horizontal transmission can occur through the ingestion of embryonated eggs found on the ground or transported on the fur of other animals. It can also occur by consuming contaminated food and infected paratenic hosts. These conditions also apply to accidental exposure in humans as paratenic hosts [7, 8].

Prevalence rates in dogs and cats have been worldwide determined mainly in coprodiagnoses ranging from 1.2% to 82.7% in dogs and 3.2%–47.2% in cats, estimating a worldwide prevalence in these domestic animals of 11.1% (95% confidence interval [CI]: 10.6%–11.7%) and 17.0% (95% CI: 16.1%–17.8%), respectively [1, 9, 10]. Regarding seroprevalence, data are scarce for cats and dogs. Only one study conducted in Iran was found, in which 53.8% seropositivity was found in 55 sampled stray cats, using the ELISA technique with crude *Toxocara cati* antigens [11]. In dogs, the highest recorded seropositivities have been observed in Iraq (71%), Brazil (82.7%), and Argentina (86.9%) [12–14].

Zoonosis associated with *Toxocara* spp. or human toxocariasis is a source of public health concern; however, it is among the five most neglected parasitic diseases according to the United States Centers for Disease Control and Prevention (CDC) [15]. This disease can be associated with different syndromes such as visceral larva migrans, ocular larva migrans, neurotoxicariasis, and/or covert toxocariasis [16–18]. Although there are experimental advances in second‐generation vaccines for its future control in dogs and cats [19, 20], only pharmacological deworming strategies are commercially available, with no biological prophylaxis options [18].

Regarding the epidemiology of this parasite in humans, Rostami et al. [21] obtained an estimated seroprevalence of 19% (95% CI: 16.6%–21.4%) for *Toxocara* spp. worldwide and 22.8% (95% CI: 19.7%–26.0%) in the Americas region. In Colombia, the ELISA technique indicated seroprevalence of 47.5% in the city of Bogotá in low‐income communities, 73.3% in the city of Villavicencio in atopic individuals, and 79.3% in an indigenous tribe in the city of Santa Marta [22–24]. ELISA is the most common serodiagnosis technique, using the excretory‐secretory products released by *T. canis* larvae (TES) [25].

Toxocariasis has been linked to the development of atopic diseases such as asthma and allergic rhinitis. This fact may be mediated by altered immune responses and chronic inflammation that this parasite can cause in human tissues, where the presence of *Toxocara* spp. larvae can stimulate the immune system leading to a modified Th2‐type response associated with high production of interleukins (IL) such as IL‐4, IL‐5, IL‐9, IL‐10, IL‐13, immunoglobulin (Ig) E and eosinophils. IL‐10 has been related to parasitic evasion that may occur against the immune system of the affected host, thus allowing high survival of the nematode in the host [26–28]. Although it is a controversial issue whether the presence of *Toxocara* spp. can generate an association in the development of allergic asthma or other atopic disease, experimental and epidemiological studies have shown a positive association in this regard. Thus, the presence of *Toxocara* spp. is a risk factor for the development of allergic asthma [29–32]. On the other hand, other studies have indicated that there was no significant association with atopic diseases [33, 34].

*Toxocara* spp. in dogs and cats has a strong impact on human health, as well as on environmental contamination, making it important to emphasize the contribution of multimodal approaches related to “One Health” to improve the control of this type of geohelminths [35, 36]. Epidemiological studies addressing *Toxocara* spp. are important for the analysis of prevalence and incidence, as well as for identifying risk factors associated with this parasitic infection. This information is essential for the development of epidemiological surveillance plans in humans and animals that lead to public health policies with a holistic approach. This activity is crucial for addressing the complex dynamics of zoonotic diseases, mainly neglected parasitic diseases [37]. The objective of the present study was to determine the seroprevalence of *Toxocara* spp. in dogs, cats, and their owners, the coprodiagnostic incidence in dogs and cats, the risk factors, and association with atopy in the owners of these pets.

## 2. Methods

### 2.1. Type of Study and Sample Size

The present longitudinal, analytical, and observational study was conducted in Villavicencio, Colombia. One whole blood sample and two fresh fecal samples were collected from each dog and cat, with a 28‐day interval between samples. The sample size was obtained through the following sampling formula (*n*) of longitudinal studies *n* = [EDFF ∗ *Np* (1 − *p*)]/[(*d*2/*Z*2 1 − *α*/2 ∗ (*N* − 1) + *p* ∗ (1 − *p*)] using the EpiInfo v.3.0 software from the United States Centers for Disease CDC (https://www.cdc.gov/epiinfo/esp/es_index.html).

The results obtained by Giraldo et al. [38] regarding the proportion of patent infection in groups of dogs from the department of Quindío, Colombia, which was 0.025, and the data obtained by Echeverry et al. [39] on patent infection by *Toxocara* spp. in cats from the same department, which indicated a proportion of 0.43, were used in the present study.

The population size of dogs and cats estimated for Villavicencio for the year 2020 was 48,000 dogs and 26,000 cats [40], using a 95% CI for the sample size calculation. A total of 70 dogs and 140 cats were sampled. Additionally, 118 adult owners of the dogs and cats that participated in the present study were linked. All owners signed an informed consent form and completed an epidemiological questionnaire following the guidelines proposed by Sherlock et al. [41], in order to determine possible risk factors associated with exposure to *Toxocara* spp.

### 2.2. Sampling and Inclusion Criteria

**FIGURE 1:**
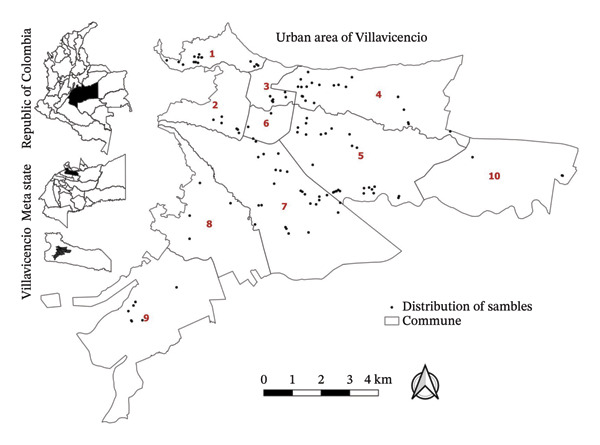
Distribution of sampling points in the ten communes of the city of Villavicencio.

Stratified random sampling was applied for the 210 domestic animals assessed in the present study (strata: 10 urban communes of the city of Villavicencio, Colombia) (Figure 1). Dogs and cats of all ages were included, as long as they had not received systemic antiparasitic agents in the last 30 days prior to sampling. Pregnant women and individuals under medical treatment were not included in the group of pet owners.

### 2.3. Serological Immunoassay in Dogs, Cats, and Owners

A total of 5 mL of blood were collected from each dog or cat and centrifuged at 2664 g for 5 minutes. The serum was separated and aliquoted for measurement of anti‐*Toxocara* spp. IgG antibodies by in‐house indirect ELISA, following the methodology standardized by Salazar Garcés et al. [19], using TES as the antigen source, obtained using the technique published by De Savigny et al. [42] and modified by Alcântara‐Neves et al. [43]. In the owners, a 5 mL blood sample was taken from the basilic, cephalic, or median veins of the anterocubital region. These samples were placed in tubes without anticoagulant and centrifuged at 2500*g* for three minutes to separate serum aliquots that were kept at −20°C until processing.

The detection of anti‐*Toxocara* IgG antibodies in the owners’ blood serum was performed using the in‐house indirect ELISA technique standardized by Jaramillo‐Hernández et al. [23]. High‐ligation 96‐well polystyrene microplates (Costar, Corning, NY, USA) were used sensitized with 3.5 μg/mL of TES diluted in carbonate/bicarbonate buffer (100 mM, pH 9.6) at 4°C overnight. After washing, the plates were blocked using a PBS solution containing 10% fetal bovine serum (FBS, Catlab, USA) and 0.05% Tween‐20 (T) for one hour at room temperature. After washing, blood sera from dogs, cats, and owners were diluted 1:1000 in a PBS‐T solution containing 2.5% FBS (PBS/T/FBS), added to the wells and incubated for 1 hour. Subsequently, the plates were washed and incubated with anticanine IgG‐peroxidase, antifeline IgG‐peroxidase, and antihuman IgG‐peroxidase conjugates for the assay for each species (Abcam®, Cambridge, England [Goat‐anti‐Dog IgG [HRP]: ab112852, Goat‐anti‐Cat IgG [HRP]: ab112801, Goat‐anti‐Human IgG [HRP]: ab6858]) at a dilution of 1:5000 for dogs and 1:10,000 for cats and owners, for one hour. A final wash was performed and the reaction was developed with 50 μL of 3,3′,5,5′‐tetramethylbenzidine (TMB) (Cell Signaling, USA) by incubating the plate in the dark for 30 min. The reaction was stopped with 50 μL/well of 2 N sulfuric acid (H2SO4). Optical density (OD) was read using the Multiskan SkyHigh absorbance microplate reader (Thermo Fisher Scientific) at a wavelength of 450 nm. Between each step, the plates were washed three times with PBS‐T and twice with PBS alone. All incubations were performed at room temperature. The cutoff point OD was 2.2085 for dogs, 1.1691 for cats, and 2.7161 for the owners, corresponding to the mean plus three standard deviations of the OD of 10 sera from the control groups for each species.

### 2.4. Coprodiagnosis of *Toxocara* spp. in Dogs and Cats

The Kato–Katz technique [44] was used to determine the number of eggs per gram of fecal matter (EPG) in samples collected from the rectal ampulla of dogs and cats, following the methodology standardized by Cárdenas Camacho et al. [45]. The selected dogs and cats that tested negative in the first sampling were considered for the second sampling (28 days later) in order to identify cumulative incidence. The latter is defined as the number of new *Toxocara* spp. positive cases in a previously negative group, adjusting the interpretation of results based on that number of eggs.

### 2.5. Skin Prick Test and International Study of Asthma and Allergies in Childhood (ISAAC) Phase III Pet Owners Survey

Extracts of the mite *Dermatophagoides pteronyssinus* 800 URC and *Blomia tropicalis* 800 URC (Alergolatina, São Paulo, Brazil), histamine chloride 1:100 (10 g/L) as a positive control, and allergen diluent (Alergolatina, São Paulo, Brazil) as a negative control were used in the skin prick test (SPT). Only one batch of allergens and controls were used throughout the study. All SPT were performed by a single observer following the methodology standardized by Jaramillo‐Hernández et al. [23]. The size of the SPT response was recorded as half the sum of the longest local inflammatory skin reaction diameter and its perpendicular diameter.

The results of SPT for allergic sensitivity to house dust mite were considered positive when the positive response to histamine was at least 3 mm, the mean of the size of the skin reactivity to the mite extract was at least 3 mm, and no positive responses to the negative saline control were recorded. Each owner completed the ISAAC III questionnaire [46], adapting the questions associated with atopic diseases. This survey is validated worldwide and allows comparisons of the prevalence of asthma, allergic rhinoconjunctivitis, and atopic eczema in adults. In addition, basic social and demographic data included in the same questionnaire were recorded [47].

### 2.6. Statistical Analysis

The data obtained from serological and coprodiagnostic analyses—in addition to the epidemiological survey and the ISAAC III survey—were initially tabulated in a database. A descriptive frequency analysis was performed for the qualitative and quantitative variables. Positive samples for *Toxocara*’s eggs in dogs and cats, in each commune, were grouped into three EPG categories (< 200, > 200 to 1000, and > 1000 EPG) and compared using the chi‐square test (*X* ^2^). Subsequently, the association of the response variables (Kato–Katz positivity and seropositivity for *Toxocara* spp.) in dogs and cats, as well as seropositivity for *Toxocara* spp. in the owners were determined, with the explanatory variables (epidemiological surveys and ISAAC III) using the chi‐square test (*X* ^2^), followed by the epidemiological indicator of prevalence ratio (PR). Then, a causal analysis (bivariate analysis) was performed with the development of a follow‐up cohort and determination of relative risk (RR) (adjusted given the prospective nature of the study design, i.e., longitudinal, analytical, and observational) and cumulative incidence (prepatent period of *Toxocara* spp. is 28 days) within the population under study through the following formula: Cumulative incidence = number of new cases of a disease during follow‐up/total population at risk at the beginning of follow‐up expressing the cumulative incidence as a percentage associated with the study time (28 days). For the different analyses, a 95% CI was taken into account, using the statistical program RStudio Version 4.5.0.

## 3. Results

### 3.1. Serology, Coprodiagnosis, and Risk Factors Associated With Dogs and Cats

**FIGURE 2:**
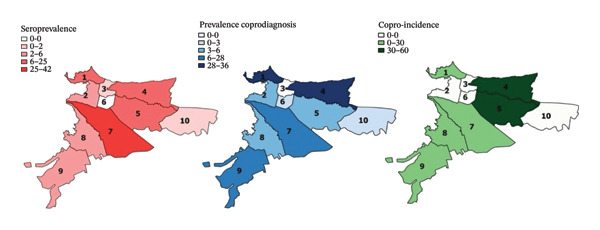
Distribution of seroprevalence, prevalence in coprodiagnosis, and coproincidence of *Toxocara* spp. in dogs by ten communes in Villavicencio.

**FIGURE 3:**
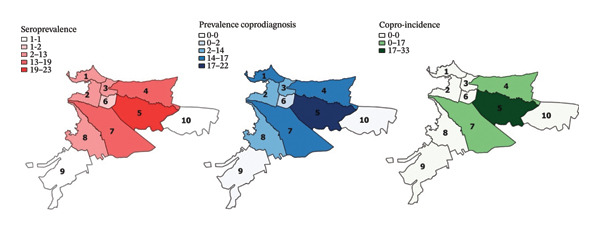
Distribution of seroprevalence, prevalence in coprodiagnosis, and coproincidence of *Toxocara* spp. in cats by ten communes in Villavicencio.

**FIGURE 4:**
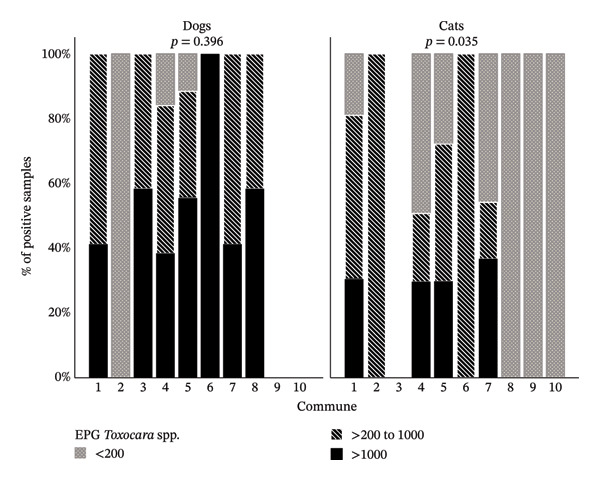
Categories of *Toxocara* spp. egg counts in feces (EPG) from dogs and cats in the ten communes of Villavicencio. *p* < 0.05, chi‐square.

The overall seroprevalence of anti‐*Toxocara* spp. IgG in dogs and cats (210 pets) was 80% (95% CI: 74.1–84.8) (168/210). It was higher in cats (85.7% [95% CI: 78.96–90.56]) than in dogs (68.6% [95% CI: 56.97–78.24]) (PR = 0.8 [95% CI 0.67–0.95]). Regarding *Toxocara* spp. seropositivity and sex, it was found that, in cats, females had a higher exposure (PR = 1.17 [95% CI: 1.01–1.34]). Additionally, regarding the seroprevalence expressed in each commune (place of residence), dogs had higher seropositivity in Commune 5 (42%), followed by Communes 4 (25%) and 7 (13%) (Figure 2). In cats, there was higher seropositivity in Commune 7 (23%), followed by Communes 4 (19%), 5 (18%), and 1 (16%) (Figure 3); however, there was no statistical association (*p* = 0.6) (Table 1). The prevalence in general coprodiagnosis was 47.6% (*n* = 100 [95% CI: 41–54.3]). The prevalence in copro‐diagnosis was higher in dogs (51.4% [95% CI: 40–62.7]) than in cats (45.7% [95% CI: 37.7–53.9]), detecting an average number EPG of 1155 and 797, respectively, for each species. The EPG count for each commune showed no significant differences in the distribution of positive dogs among the ten communes evaluated (*X* ^2^ = 14.7 and *p* = 0.396). In contrast, the distribution of EPG categories in positive cats varied significantly among the communes (*X* ^2^ = 27.6 and *p* = 0.035) (Figure 4). Commune 7, followed by Communes 4, 5, and 1, had the highest proportion of cats with high parasite loads (> 1000 EPG).

**TABLE 1:** Characterization of dogs and cats according to their positivity for ELISA and Kato–Katz for *Toxocara* spp. in Villavicencio.

Variable	Category	Seropositive (%)	Seronegative (%)	Relative frequency (%)	*X* ^2^	PR (95% CI)	*p* value
		168 (80)	42 (20)				
Species	Canines	48 (28.6)	22 (52.4)	33.3	8.57	0.8 (0.67–0.95)	^∗^0.003
	Felines	120 (71.4)	20 (47.6)	66.7			

Dogs	Kato–Katz (+)	36 (21.4)	0 (0)	17.1	N/A		
	Kato–Katz (−)	12 (7.14)	22 (52.4)	16.2			

Cats	Kato–Katz (+)	64 (38.1)	0 (0)	30.5	N/A		
	Kato–Katz (−)	56 (33.4)	20 (47.6)	36.2			

Dog sex	Females	34 (20.2)	13 (30.9)	22.4	0.94	1.19 (0.82–1.72)	0.33
	Males	14 (8.4)	9 (21.4)	11			

Sex cats	Females	68 (40.5)	6 (14.3)	35.2	4.89	1.17 (1.01–1.34)	^∗^0.02
	Males	52 (30.9)	14 (33.4)	31.4			

Age	0–5	92 (54.2)	30 (71.4)	58.1	4.64	1	0.098
	6–10	61 (36.9)	8 (19.1)	32.9		1.17 (1.03–1.34)	
	11–15	15 (8.9)	4 (9.5)	9		1.05 (0.81–1.35)	

Commune (area of residence)	1	22 (13.1)	6 (14.3)	13.3	7.35	1	0.6
	2	7 (4.2)	1 (2.4)	3.8		1.11 (0.80–1.54)	
	3	4 (2.4)	3 (7.1)	3.3		0.73 (0.37–1.42)	
	4	35 (20.8)	7 (16.7)	20		1.1 (0.84–1.34)	
	5	42 (25)	10 (23.8)	24.8		1.01 (0.81–1.3)	
	6	2 (1.2)	0 (0)	1		1.27 (1.05–1.54)	
	7	33 (19.6)	8 (19)	19.5		1.02 (0.8–1.31)	
	8	6 (3.6)	3 (7.1)	4.3		0.85 (0.51–1.4)	
	9	15 (8.9)	2 (4.8)	8.1		1.12 (0.87–1.46)	
	10	2 (1.2)	2 (4.8)	1.9		0.64 (0.23–1.73)	

Abbreviations: 95% CI, confidence interval; N/A, not applicable; PR, prevalence ratio.

^∗^Statistically associated.

Of the 210 animals studied, only 110 that tested negative in the first sample (34 dogs and 76 cats) were considered for the second fecal sample. The coprodiagnosis indicated an overall cumulative incidence of 20% (*n* = 22 [95% CI: 13.6–28.4]), with a higher incidence rate in dogs (29.4% [*n* = 10; 95% CI: 16.83–46.2]) than in cats (15.8% [*n* = 12; 95% CI: 9.3–25.6]) (Table 2).

**TABLE 2:** Identification of risk factors associated with *Toxocara* spp. in dogs and cats in Villavicencio.

Variable	Category	Positive (%)	Negative (%)	Relative frequency (%)	*X* ^2^	*p* value	RR (95% CI)
		22 (20)	88 (80)				
Species	Canine	10 (45.4)	24 (27.3)	30.9	2.72	0.098	1.86 (0.89–3.89)
	Feline	12 (54.5)	64 (72.7)	69.1			

Sex	Female	10 (45.4)	52 (59.1)	56.4	1.33	0.248	0.65 (0.30–1.36)
	Male	12 (54.5)	36 (40.9)	43.6			

Age	< 3 years	5 (22.7)	32 (36.4)	33.6	1.86	0.393	1
	3–8 years	12 (54.6)	35 (39.8)	42.7			1.88 (0.73–4.88)
	9–16 years	5 (22.7)	21 (23.8)	23.7			1.42 (0.45–4.42)

Socioeconomic level	Low	8 (36.4)	28 (31.8)	32.7	0.17	0.684	1.17 (0.54–2.54)
	Medium	14 (63.6)	60 (68.2)	67.3			

Commune (area of residence)	1	1 (4.5)	11 (12.5)	10.9	N/A		
	2	0 (0)	4 (4.5)	3.6			
	3	0 (0)	5 (5.7)	4.5			
	4	4 (18.2)	14 (16)	16.4			
	5	10 (45.6)	19 (21.6)	26.4			
	6	0 (0)	0 (0)	N/A			
	7	5 (22.7)	23 (26.1)	25.5			
	8	1 (4.5)	4 (4.5)	4.5			
	9	1 (4.5)	5 (5.7)	5.5			
	10	0 (0)	3 (3.4)	2.7			

When was your pet last dewormed?	< 3 months	3 (13.6)	15 (17)	16.4	4.22	0.121	1
	3–6 months	14 (63.7)	35 (39.8)	44.5			1.71 (0.56–5.27)
	> 6 months	5 (22.7)	38 (43.2)	39.1			0.69 (0.18–2.61)

Vaccination for your pet?	Not > 2 years	2 (9.2)	11 (12.5)	11.8	3.53	0.17	0.42 (0.12–1.95)
	Full annual reinforcement	10 (45.4)	22 (25)	29.1			1
	Annual antirabies vaccination only	10 (45.4)	55 (62.5)	59.1			0.49 (0.22–1.06)

Type of power supply?	Home‐cooked diet		2 (2.2)	1.8	8.73	0.635	0
	Balanced commercial food	10 (45.4)	44 (50)	49.1			1
	Mixed	12 (54.6)	42 (47.8)	49.1			1.2 (0.56–2.53)

What kind of water does your pet drink?	Potable water	8 (36.4)	23 (26.1)	28.2	0.90	0.34	1.46 (0.67–3.12)
	Nonpotable water	14 (63.6)	65 (73.9)	71.8			

Where does your pet sleep?	Sleeps with the owner	15 (68.1)	48 (54.5)	57.3	1.32	0.515	1.61 (0.58–4.39)
	Own bed	4 (18.2)	23 (26.2)	24.5			1
	Another area	3 (13.7)	17 (19.3)	18.2			1.01 (0.25–4.02)

Where does your pet defecate?	Indoor	5 (22.7)	52 (59.1)	51.8	9.32	^∗^0.0023	0.27 (0.10–0.69)
	Outdoor	17 (77.3)	36 (40.9)	48.2			

How many times a day do you take your pet out?	< 3 times	3 (13.7)	14 (15.9)	15.5	10.02	^∗^0.006	1.9 (0.50–7.15)
	> 3 times	14 (63.6)	25 (28.4)	35.4			3.87 (1.52–9.87)
	No outdoor access	5 (22.7)	49 (55.7)	49.1			1

How many people live in the household?	< 3	16 (72.7)	47 (53.4)	57.3	2.68	0.101	1.99 (0.84–4.69)
	> 3	6 (27.3)	41 (46.6)	42.7			

Does your pet live with other types of animals?	Yes	16 (72.7)	59 (67)	68.2	0.26	0.608	1.24 (0.34–1.87)
	No	6 (27.3)	29 (33)	31.8			

Abbreviations: 95% CI, confidence interval; Mixed, home‐cooked diet and balanced commercial food; N/A, not applicable; RR, relative risk.

^∗^Statistically associated.

In the bivariate model (Table 2), regarding age, a higher seroprevalence was found in dogs and cats aged three to eight years compared to those aged under three years or nine to sixteen years although the differences were not statistically significant (*p* = 0.393). Epidemiological data showed that if a pet defecated inside the home, it was 3.7 times less likely to contract *Toxocara* spp. than pets that defecated outside (RR = 0.27; 95% CI: 0.10–0.69; and *p* = 0.0023). Pets that went outside more than three times a day were more likely to contract *Toxocara* spp. than pets that did not go outside or went outside less than three times (RR = 3.87; 95% CI: 1.52–9.87; and *p* = 0.006).

### 3.2. Serology and Associated Risk Factors in Pet Owners

**FIGURE 5:**
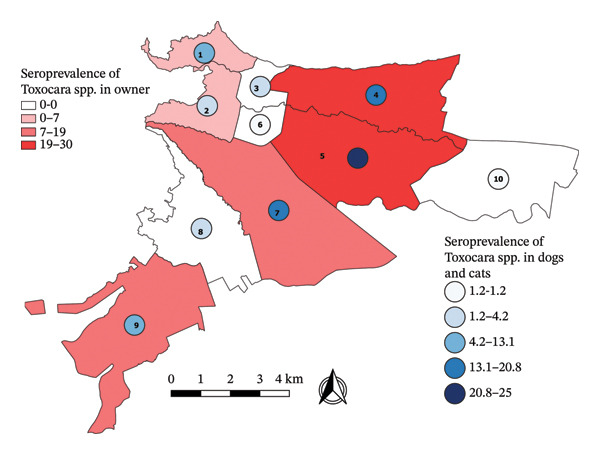
Distribution of *Toxocara* spp. seroprevalence in pet owners, dog, and cat by communes in Villavicencio.

The overall seroprevalence of anti‐*Toxocara* IgG in humans (*n* = 118) was 22.9% (*n* = 27 [95% CI: 16.2–31.2]). Regarding age, sex, socioeconomic level, and area of residence (commune), there was no statistically significant relationship with *Toxocara* spp. Infection (*p* ≥ 0.05), although individuals of low socioeconomic level, tended to be more seroprevalent (*p* = 0.06). Among pet owners, the highest seropositivities were found in Communes 4 (26%) and 5 (29.6%), followed by Communes 7 (18.5%) and 9 (11.1%) (Figure 5).

Regarding clinical variables related to the ISAAC III survey and SPT results, *Toxocara* spp. seropositivity in the owners was not significantly associated with atopy (*p* = 0.78). Individuals who owned both a seropositive cat and a seropositive dog at the same time were more likely to be seropositive than those who owned either a *Toxocara* spp. seropositive cat or dog (PR = 3.46 [95% CI: 1.9–6.3]) (Table 3).

**TABLE 3:** Characterization of pet owners, their serodiagnostic to *Toxocara* spp., and atopic conditions in Villavicencio.

Variable	Category	Seropositive (%)	Seronegative (%)	Relative frequency (%)	*X* ^2^	PR (95% CI)	*p* value
		27 (22.9)	91 (77.1)				
Age	30–59 years	7 (25.9)	26 (28.6)	28	0.88	1.00	0.64
	18–29 years	19 (70.4)	64 (70.3)	70.3		1.08 (0.50–2.32)	
	> 60 years	1 (3.7)	1 (1.1)	1.7		2.36 (0.50–10.93)	

Sex	Female	18 (66.7)	63 (69.2)	68.6	0.06	0.91 (0.45–1.83)	0.80
	Male	9 (33.3)	28 (30.8)	31.4			

Socioeconomic level	Low	12 (44.4)	23 (25.3)	29.7	3.67	1.90 (0.99–3.62)	0.06
	Medium	15 (55.6)	68 (74.7)	70.3			

Commune (area of residence)	1	2 (7.4)	14 (15.4)	13.6	9.76	1	0.36
	2	2 (7.4)	4 (4.4)	5.1		2.66 (0.48–14.9)	
	3	0 (0)	6 (6.6)	5.1		0	
	4	7 (26)	15 (16.5)	18.6		2.54 (0.61–10.67)	
	5	8 (29.6)	17 (18.7)	21.2		2.56 (0.62–10.56)	
	6	0 (0)	2 (2.2)	1.7		0	
	7	5 (18.5)	18 (19.8)	19.5		1.74 (0.38–7.88)	
	8	0 (0)	7 (7.7)	5.9		0	
	9	3 (11.1)	5 (5.5)	6.8		3 (0.62–14.49)	
	10	0 (0)	3 (3.3)	2.5		0	

Asthma	Yes	2 (7.4)	11 (12.1)	11	0.47	0.65 (0.17–2.42)	0.50
	No	25 (92.6)	80 (87.9)	89			

Rhinitis	Yes	13 (48.1)	46 (50.5)	50	0.048	0.93 (0.47–1.8)	0.86
	No	14 (51.9)	45 (49.5)	50			

Atopic dermatitis	Yes	7 (25.9)	35 (38.5)	35.6	1.427	0.63 (0.29–1.37)	0.232
	No	20 (74.1)	56 (61.5)	64.4			

SPT	Yes	13 (48.1)	36 (39.6)	41.5	0.63	1.31 (0.67–2.53)	0.426
	No	14 (51.9)	55 (60.4)	58.5			

Atopy	Yes	22 (48.1)	72 (39.6)	79.7	0.07	1.02 (0.83–1.26)	0.78
	No	5 (18.5)	19 (39.6)	20.3			

Dog (+)	Yes	5 (18.5)	22 (24.2)	22.9	0.37	0.76 (0.32–1.83)	0.54
	No	22 (81.5)	69 (75.8)	77.1			

Cat (+)	Yes	16 (59.3)	48 (52.7)	54.2	0.35	1.23 (0.62–2.41)	0.55
	No	11 (40.7)	43 (47.3)	45.8			

Dog and cat (+)	Yes	6 (22.2)	3 (3.3)	7.6	10.58	3.46 (1.9–6.3)	^∗^0.001
	No	21 (77.8)	88 (96.7)	92.4			

*Note:* (+) = *Toxocara* spp. pet seropositive.

Abbreviations: 95% CI, confidence interval; N/A, not applicable; PR, prevalence ratio; SPT, skin prick test.

^∗^Statistically associated.

The bivariate analysis indicated that owning a dog was a protective factor. Owning a dog generated a 2.1 times lower risk of being seropositive for *Toxocara* spp. than owning a cat (RR = 0.48; 95% CI: 0.29–0.76; and *p* = 0.0001). Owning a pet aged nine to 16 years generated a higher risk of exposure to *Toxocara* spp. than having pets aged under 8 years (RR = 1.5; 95% CI: 1.03–2.18; and *p* = 0.042). It was also found that pets that slept in their owners’ beds generated a higher risk to these owners of being seropositive to *Toxocara* spp. than when the pets slept in their own beds or other areas (RR = 1.93; 95% CI: 1.15–3.23; and *p* = 0.0087) (Table 4).

**TABLE 4:** Identification of risk factors associated with *Toxocara* spp. in pet owners in Villavicencio.

Variable	Category	Positive (%)	Negative (%)	Relative frequency (%)	*X* ^2^	*p* value	RR (95% CI)
		68 (62)	42 (38)				
Species	Canine	12 (17.6)	22 (52.4)	30.9	14.667	^∗^0.0001	0.48 (0.30–0.76)
	Feline	56 (82.4)	20 (47.6)	69.1			

Sex	Female	43 (63.2)	19 (45.2)	56.4	4.419	0.064	1.33 (0.97–1.83)
	Male	25 (36.8)	23 (54.8)	43.6			

Age	< 3 years	19 (27.9)	18 (42.9)	33.6	4.19	^∗^0.042	1
	3–8 years	29 (42.6)	18 (42.9)	42.7			1.2 (0.82–1.77)
	9–16 years	20 (29.5)	6 (14.2)	23.7			1.5 (1.03–2.18)

Socioeconomic level	Low	23 (33.8)	13 (31)	32.7	0.09	0.755	1.05 (0.77–1.42)
	Medium	45 (66.2)	29 (69)	67.3			

Commune (area of residence)	1	6 (8.8)	6 (14.3)	10.9	N/A		
	2	3 (4.4)	1 (2.4)	3.6			
	3	2 (2.9)	3 (7.1)	4.5			
	4	11 (16.2)	7 (16.7)	16.4			
	5	19 (28)	10 (23.8)	26.4			
	6	0 (0)	0 (0)	0			
	7	20 (29.4)	8 (19)	25.5			
	8	2 (2.9)	3 (7.1)	4.5			
	9	4 (5.9)	2 (4.8)	5.5			
	10	1 (1.5)	2 (4.8)	2.7			

When was your pet last dewormed?	< 3 months	9 (13.2)	9 (21.4)	16.4	1.633	0.441	1
	3–6 months	30 (44.1)	19 (45.2)	44.5			1.35 (0.81–2.24)
	> 6 months	29 (42.7)	14 (33.4)	39.1			1.22 (0.73–2.04)

Where does your pet sleep?	Sleeps with the owner	45 (66.2)	18 (42.8)	57.3	9.488	^∗^0.0087	1.93 (1.15–3.23)
	Own bed	10 (14.7)	17 (40.5)	24.5			1
	Another area	13 (19.1)	7 (16.7)	18.2			1.75 (0.97–3.16)

Where does your pet defecate?	Indoor	34 (50)	23 (54.8)	51.8	0.235	0.627	0.93 (0.69–1.24)
	Outdoor	34 (50)	19 (45.2)	48.2			

How many times a day do you take your pet out?	< 3 times	9 (13.2)	8 (19)	15.5	0.958	0.619	0.86 (0.52–1.42)
	> 3 times	26 (38.3)	13 (31)	35.4			1.09 (0.80–1.48)
	No outdoor access	33 (48.5)	21 (50)	49.1			1

How many people live in the household?	< 3	40 (58.8)	23 (54.8)	57.3	0.175	0.675	1.07 (0.78–1.43)
	> 3	28 (41.2)	19 (45.2)	42.7			

Does your pet live with other types of animals?	Yes	47 (69.1)	28 (66.7)	68.2	0.072	0.788	1.04 (0.75–1.44)
	No	21 (30.9)	14 (33.4)	31.8			

Abbreviations: 95% CI, confidence interval; N/A, not applicable; RR, relative risk.

^∗^Statistically associated.

## 4. Discussion

Most studies emphasize the prevalence of *Toxocara* spp. in dogs and cats through coprodiagnoses [1]. However, to date, no seroepidemiological studies have been conducted in Colombia addressing the prevalence of this parasite in domestic animals. This is the first study addressing the seroprevalence of *Toxocara* spp. in dogs and cats in the country, finding a high overall seroprevalence rate in pets, being higher in cats than in dogs (Table 1). The seroprevalence observed in dogs was higher than that reported by Nandini et al. [48] who recorded a seroprevalence of 39.6% in India. Also, in Argentina, Rubel et al. [49] found seroprevalence of 22% and 40% in low‐ and middle‐income regions, respectively. In Mexico, Tinoco‐Gracia et al. [50] and Martínez‐Barbabosa et al. [51] found seroprevalence of 56.1% and 66.7%, respectively. The highest seroprevalence rate has been reported in dogs in Argentina (86.9%), followed by Brazil (82.7%) and Iraq (71%) [12–14]. Regarding domestic cats, the present study presents the highest seroprevalence rate compared with 53.8% found by Meshgi et al. [11].

The prevalence of *Toxocara* spp. in coprodiagnoses in dogs and cats varies widely worldwide, with values ranging from 0.9% to 94.4% [6, 52]. The present study reports the highest coprodiagnostic prevalence rates in Colombia in dogs and cats, compared with the results reported by López‐Arias et al. [53], who found a prevalence of 5.6% in dogs and 2.5% in cats, Sarmiento‐Rubiano et al. [54], with a prevalence of 12.4% in dogs and 8.9% in cats, and Peña‐Quistial et al. [55], with a prevalence of 25% in dogs and 44% in cats, using the Kato–Katz technique.

The prevalence in both coprodiagnoses and immunoassays were higher in adult dogs and cats (Tables 1 and 2). *Toxocara* spp. can generate a state of hypobiosis [1], causing the larvae to remain dormant in different tissues of the host. In addition, adult animals have a greater exposure to sources of infection, and although adult dogs develop a humoral response, they may remain susceptible to infection by this geohelminth [56]. These findings are consistent with the results obtained by Jarad et al. [12] and Regis et al. [13], according to which older dogs were more seropositive than younger ones. Although in the present study there was no statistically significant difference, this fact was characterized as a risk factor for toxocariasis in pet owners (Table 4).

In the present study, the frequency of outdoor activities and defecation outside the home were found to be significant risk factors in dogs and cats (Table 2). Longer outdoor exposure increases the risk of pets developing patent infections, mainly due to the possibility of ingesting infectious eggs from the environment or through their predatory behavior of paratenic hosts regarding cats [57, 58]. Pets that roam freely or are frequently walked daily can contaminate the environment with *Toxocara* spp. eggs through their feces, leading most public places, such as parks, to be contaminated with this geohelminth [59–61].

*Toxocara* spp. infection is one of the most prevalent zoonotic helminthiases globally due to its resistance and high reproductive capacity [1]. It is worth mentioning that the environmental conditions of tropical and subtropical areas favor the survival of eggs, which generates a greater exposure to contracting toxocariasis [2]. Colombia, at the level of South America, has stood out for having high seroprevalence rates in humans [62], with 47.5% in Bogotá, 73.3% in Villavicencio, and 79.3% in Santa Marta [22–24]. The seroprevalence found in the present study was lower than that reported in those studies (Table 3).

Individuals with a low socioeconomic level have greater difficulty accessing adequate health services and veterinary care. In addition, hygiene and housing conditions may be poor, which can facilitate contact with environments contaminated by *Toxocara* spp., especially in urban environments [63, 64]. However, in the present study, there was no statistically significant difference with respect to *Toxocara* spp. seropositivity and socioeconomic status (Table 4). Individuals with a low socioeconomic status in the present study tended to be more seropositive, and this can also be extrapolated to domestic animals since dogs have been found to be more prevalent in low‐income households [49]. It is important to underline this finding with the need to reduce inequality in public health in order to reduce the prevalence of zoonotic diseases and contribute to the One Health concept [65].

Regarding place of residence, the most seroprevalent areas for dogs, cats, and pet owners were Communes 4 and 5 (Figure 5), and according to the EPG per commune in dogs and cats, the parasite load was heterogeneous in cats, unlike in dogs (Figure 4). These variations point to possible local socioeconomic and environmental determinants, such as soil contamination, dog and cat feces management or access to veterinary services. However, no statistically significant association was found between geographical location by commune and seropositivity to *Toxocara* spp. These variations are related to similar patterns in other regions of the world, where the prevalence of this parasite varies considerably depending on climatic‐environmental conditions [60], socioeconomic level, urban density, and coexistence with other domestic animals or with stray animals [6, 9, 21].

In the present study, no significant association was found between *Toxocara* spp. seroprevalence in humans and atopy according to the ISAAC III survey and the SPT. These results agree with those reported by Jaramillo‐Hernández et al. [23] in the same city and with those reported by De Sanctis et al. [33] and Pouryousef et al. [34], in which there was no significant association between *Toxocara* spp. seroprevalence and atopic disease. Studies have found an inverse association with respect to the SPT. This fact could be explained by different mechanisms, such as a high production of polyclonal IgE induced by *Toxocara* spp. and nonspecific for aeroallergens, hindering histamine release and thus reducing test reactivity, blockage by IgG4 that competes with IgE for binding to allergens, and an immunomodulatory response with the production of IL‐10 and other anti‐inflammatory cytokines that increase mast cell activation even when allergen‐specific IgE is present [31, 66]. These mechanisms could also be related to the low association with asthma and atopic rhinitis [29, 67].

As a risk factor associated with exposure to *Toxocara* spp., seropositivity for this parasite in dogs and cats is highly correlated with seropositivity in humans, especially in young individuals [68]. This fact is consistent with the results obtained in the present study, which indicated higher human seropositivity when a seropositive dog and a seropositive cat lived in the same household (Table 3). Accidental ingestion of *Toxocara* spp. eggs in the environment is the main route of transmission, with public places such as parks where individuals often take their pets to defecate, reflecting a potential risk of exposure for those who live in close contact with them [69]. Additionally, the present study found another risk factor, i.e., sleeping in the same bed with the pets (Table 4), since the presence of eggs of this parasite has been found in the coats of dogs and in the shoes of their owners, thus reaffirming the potential for exposure to this zoonotic parasite [70, 71].

Globally, according to meta‐analyses, an estimated prevalence of *Toxocara* spp. of 11.1% and 17% has been observed in dogs and cats, respectively [6, 9]. The present study found that owning a dog was a protective factor that reduced the risk of developing *Toxocara* spp. (Table 4). Since young or stray dogs may be more vulnerable, their prevalence is lower in urban areas and domestic environments, where deworming protocols are stricter [58]. In contrast, cats have exhibited high parasitic loads, even domestic cats, indicating that they can release eggs into the environment more effectively due to their natural behavior, hunting instinct, and tendency to explore areas, increasing their exposure to the parasite and generating greater zoonotic risk. This fact clearly demonstrates the need to prioritize antiparasitic control in this species [55, 72, 73].

Among the limitations of the present study, the ELISA test implemented in pets did not differentiate between *T. canis* and *T. cati* infections, since they share similar surface and excretory‐secretory antigens (TES) [74, 75]. Likewise, the diagnoses were based exclusively on ELISA, without the application of additional confirmatory tests to rule out cross‐reactions with other helminths (e.g., *Ancylostoma* spp.). These limitations could have influenced the estimated prevalence in the pets and their owners and should, therefore, be considered when interpreting the results, although previous studies have indicated that the use of ELISA with TES antigen to diagnose toxocariasis in humans was widely used [21, 62]. However, in tropical areas, high cross‐reactivity with helminths has been observed, especially with the genus *Ascaris* in humans [76, 77].

Nonetheless, the use of ELISA with recombinant TES antigens, which improves specificity, is recommended for further studies [76]. This analysis is accompanied by an avidity test that allows identifying antibody affinity, targeting the phase of infection. Also, Western blotting allows identifying specific antibodies against parasite proteins, since TES antigens present epitopes that cross‐react with other parasites [70, 78, 79]. Additionally, the preabsorption of serum with *Ascaris suum* or *Ascaris lumbricoide* in humans can be considered to reduce false‐positive results [80, 81].

## 5. Conclusion

The findings of the present study demonstrated a high prevalence of *Toxocara* spp. in dogs and cats and its significant association between positivity and access and frequency of going outside the homes. This fact suggests the need to reinforce periodic deworming programs for dogs and cats, especially in urban areas with a high density of these pets, as well as restricting access to contaminated public areas [1].

In humans, there was a low seropositivity and the main risk factors observed highlight the importance of performing informative interventions to improve hygienic‐sanitary measures, integrating a multidisciplinary approach under the One Health concept, which considers both human health and animal health. These results support the integration of *Toxocara* spp. in epidemiological surveillance programs for this type of neglected geohelminths, mainly in tropical regions where there are high rates of parasitic infections.

## Author Contributions

María Alejandra Velásquez Peña: methodology, investigation, visualization, writing–original draft preparation, and writing–review and editing. Chaira Liz Angelica Vasquez‐Turriago: writing–review and editing. Adolfo Vásquez‐Trujillo: data curation, formal analysis, and validation. Dumar Alexander Jaramillo‐Hernández: conceptualization, formal analysis, funding acquisition, methodology, project administration, investigation, supervision, visualization, writing–original draft preparation, and writing–review and editing.

## Funding

The Directorate General for Research at the University of the Llanos (Colombia), through the Call for Proposals to Strengthen Research Groups Categorized as A1, A, and B–Call 894 of 2021 minCiencias, provided financial support for the project: Incidence of *Toxocara* spp. in companion animals and its possible impact on public health in the municipality of Villavicencio, code C09‐F01‐001‐2022.

## Disclosure

All authors have read and approved the final version of the manuscript for submission.

## Ethics Statement

This research was endorsed by the Bioethics Committee of the University of the Llanos, according to Act 005 by consensus on 1 September 2022. In addition, all owners of dogs and cats involved in this study signed the respective informed consent form.

## Conflicts of Interest

The authors declare no conflicts of interest.

## Data Availability

The data that support the findings of this study are available from the corresponding author upon reasonable request.
